# Supplementary material for: Causal Relationship between Aging and Anorexia Nervosa: A White-Matter-Microstructure-Mediated Mendelian Randomization Analysis
Source: Biomedicines. 2024 Aug 16;12(8):1874. doi: 10.3390/biomedicines12081874 (PMC11351342; doi:10.3390/biomedicines12081874)
Supplement: Supplementary file 1 [file biomedicines-12-01874-s001.zip › Supplementary Figure/Figure S8/Leaveoneout plot of MR analysis for TL to ubm-b-1742.pdf]

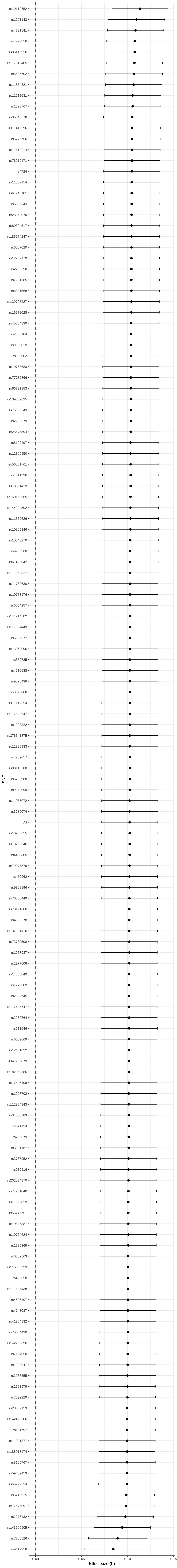
rs10112752
rs1291143
rs4731541
rs7790856
rs35446936
rs117512405
rs6536702
rs11584821
rs11212631
rs1023767
rs35640778
rs11412296
rs6776756
rs12412214
rs76219171
rs4724
rs11557154
rs61748181
rs6590343
rs28363070
rs80324517
rs185174247
rs6007020
rs12932179
rs2230590
rs7221585
rs6881568
rs139795227
rs16978028
rs45604339
rs2555104
rs9600019
rs932002
rs10768683
rs77732866
rs66731853
rs139669835
rs76065543
rs2293579
rs28577594
rs8102497
rs12369950
rs56061761
rs1611236
rs73581419
rs150150565
rs144204502
rs11579626
rs10805346
rs10840270
rs9955360
rs61405042
rs111950327
rs11769630
rs10773176
rs6054257
rs141214782
rs117034449
rs6587577
rs13062095
rs869785
rs4616688
rs9878436
rs3093888
rs11117354
rs117630647
rs1003322
rs376641875
rs12925933
rs7209057
rs80116508
rs6790988
rs9940099
rs11085072
rs3785074
All
rs10905255
rs13230646
rs4498805
rs79977579
rs450962
rs9398196
rs76666449
rs78491606
rs4530278
rs137901416
rs73730598
rs1957937
rs2977608
rs17803849
rs7772289
rs2538745
rs117407747
rs2282764
rs611646
rs6659669
rs12451892
rs41269079
rs182059586
rs17445108
rs1907702
rs112394943
rs34550383
rs871134
rs762679
rs3891167
rs3767952
rs939916
rs202034370
rs77231040
rs11699829
rs55747751
rs10845387
rs10774624
rs1985369
rs6669563
rs116863223
rs429358
rs111527438
rs4695407
rs4743037
rs41304832
rs75664430
rs142730696
rs7164950
rs1332941
rs2967355
rs2763979
rs7099229
rs28502153
rs142426306
rs131797
rs11991877
rs188918174
rs8105767
rs59409453
rs56799554
rs5742915
rs17677991
rs2276182
rs143190905
rs7705526
rs9419958
SNP
0.00
0.05
0.10
0.15
Effect size (b)
